# Human *Angiostrongylus cantonensis*, Jamaica

**DOI:** 10.3201/eid1112.050217

**Published:** 2005-12

**Authors:** Cecelia A. Waugh, Shira Shafir, Matthew Wise, Ralph D. Robinson, Mark L. Eberhard, John F. Lindo

**Affiliations:** * University of West Indies, Kingston, Jamaica; † Centers for Disease Control and Prevention, Atlanta, Georgia, USA

**Keywords:** Angiostrongylus cantonensis, zoonoses, Jamaica, letter

**To the Editor:** *Angiostrongylus cantonensis* is the most common cause of eosinophilic meningoencephalitis worldwide (*1*). The parasite's presence has been well documented in Jamaica in rats (definitive host) and a variety of mollusks (intermediate hosts); infections occur in humans sporadically on the island. However, the mode of transmission of infections to humans in Jamaica, where raw or undercooked mollusks are not usually eaten, is not well understood (*2*).

An outbreak of *A. cantonensis* occurred among American medical students vacationing in Jamaica in 2000. An epidemiologic investigation identified the probable source of infection (Caesar salad), but no biologic contaminant was determined (*2*). During a field investigation of *A. cantonensis*, we spoke with local farmers and vendors to identify possible routes of food contamination. While our observations were preliminary and anecdotal in nature, our findings provide valuable insight into local transmission and control of this parasite.

Humans can become infected by eating the intermediate hosts, slugs and snails, of *A. cantonensis*. Freshwater shrimp serve as paratenic hosts and reservoirs of infection for humans, both naturally and experimentally (*3**,**4*). Most reports of Jamaican eating practices indicate that terrestrial snails and slugs are not eaten and that shrimp and other meats are always eaten well cooked (*5*). However, during interviews with a farmer near Mavis Bank, a rural area outside of Kingston, and fishermen at the Coronation Market, Jamaica's largest fresh produce market, we discovered that freshwater and saltwater shrimp, as well as mussels (paratenic hosts), are occasionally eaten raw. Freshwater shrimp or mussels are eaten, particularly by men, directly from rivers and streams, and freshwater and saltwater bait shrimp are eaten by fishermen.

In Jamaica, molluscicides are routinely applied to growing vegetables such as cabbage, lettuce, and bok choy to keep snails and slugs away, although this practice is not effective. Snails and slugs withdrew from produce after the molluscicide was applied to surrounding vegetation, but returned after several days. We purchased a lettuce head that had been reportedly treated with molluscicides at the Coronation Market and found a small slug inside. The role of produce in transmitting *A. cantonensis* is still unclear; humans may become infected by inadvertently consuming small slugs or other infected hosts or by consuming produce directly contaminated with larvae. Infections in slugs have not been found in previous studies conducted on the island (*2*). Regardless, the use of molluscicides to limit human infection from produce is an ineffective strategy.

At the Coronation Market, vendors repeatedly used a bucket of water to rinse vegetables before displaying them. This practice could transmit *A. cantonensis* in 2 ways. First, if free larvae are deposited on vegetables in either the slime or feces of mollusks, cross contamination can occur. Second, dead or decaying intermediate hosts may release larvae into water (*6*). If infected mollusks were rinsed from vegetables into the buckets, the water could become contaminated with larvae. While cross-contamination by common wash buckets has not been implicated in an outbreak of a parasitic infection, it has been linked to outbreaks of other infectious agents (*7**,**8*).

Vendors at venues such as Coronation Market primarily buy produce to sell. These vendors typically purchase their produce from intermediaries who purchase and transport it from farms in outlying areas. As a consequence, many vendors are unsure of the farm or region from which their produce came. This practice makes it difficult, if not impossible, for health officials and researchers to isolate and link etiologic agents with particular produce items or regions and complicates the investigation of any foodborne infection.

*A. cantonensis* is an important parasitic agent in Jamaica for which a definitive route of infection is often not found. We found that potential paratenic hosts are occasionally eaten raw. Because of the high prevalence of *A. cantonensis* infection in mollusks in certain parts of Jamaica, consumption of raw, infected shrimp may be a source of sporadic angiostrongyliasis on the island. Control of *A. cantonensis* is complicated because of the apparent ineffectiveness of molluscicides, the potential for cross-contamination of produce at markets, and the difficulty of tracking produce and other products to their source.

## References

[1] Kliks MM, Palumbo NE. Eosinophilic meningitis beyond the Pacific Basin: the global dispersal of a peridomestic zoonosis caused by *Angiostrongylus cantonensis*, the nematode lungworm of rats. Soc Sci Med. 1992;34:199–212. 10.1016/0277-9536(92)90097-A 1738873

[2] Lindo JF, Waugh C, Hall J, Cunningham-Myrie C, Ashley D, Eberhard ML, Enzootic *Angiostrongylus cantonensis* in rats and snails after an outbreak of human eosinophilic meningitis, Jamaica. Emerg Infect Dis. 2002;8:324–6. 10.3201/eid0803.010316 11927033 PMC2732477

[3] Alto W. Human infections with *Angiostrongylus cantonensis.* Pac Health Dialog. 2001;8:176–82. 12017820

[4] Wallace GD, Rosen L. Studies on eosinophilic meningitis. 2. Experimental infection of shrimp and crabs with *Angiostrongylus cantonesnsis.* Am J Epidemiol. 1966;84:120–31. 5944728 10.1093/oxfordjournals.aje.a120617

[5] Lindo JF, Escoffery CT, Reid B, Codrington G, Cunningham-Myrie C, Eberhard ML. Fatal autochthonous eosinophilic meningitis in a Jamaican child caused by *Angiostrongylus cantonensis.* Am J Trop Med Hyg. 2004;70:425–8. 15100458

[6] Cross JH. Angiostrongylosis. In: Palmer SR, Soulsby EJL, Simpson DIY, editors. Zoonoses. Oxford: Oxford University Press; 1998. p. 774–81.

[7] Centers for Disease Control and Prevention. Hepatitis A outbreak associated with green onions at a restaurant—Monaca, Pennsylvania, 2003. MMWR Morb Mortal Wkly Rep. 2003;52:1155–7. 14647018

[8] Wachtel MR, Charkowski AO. Cross-contamination of lettuce with *Escherichia coli* O157:H7. J Food Prot. 2002;65:465–70. 11899044 10.4315/0362-028x-65.3.465

